# Comparative Analysis of Severe Clinical Outcomes in Hospitalized Patients with RSV, Influenza, and COVID-19 Across Early and Late COVID-19 Pandemic Phases (2021–2024)

**DOI:** 10.3390/jcm14144894

**Published:** 2025-07-10

**Authors:** Yuying Xing, Amit Bahl

**Affiliations:** 1 Corewell Health Research Institute, Royal Oak, MI 48073, USA; yuying.xing@corewellhealth.org; 2 Department of Emergency Medicine, Corewell Health William Beaumont University Hospital, Mile Rd, Royal Oak, MI 48073, USA; 3 Oakland University William Beaumont School of Medicine, Rochester, MI 48309, USA

**Keywords:** COVID-19, RSV, influenza, severe outcomes, viral infections, respiratory illnesses

## Abstract

**Background:** COVID-19, influenza, and respiratory syncytial virus (RSV) are major respiratory infections with overlapping clinical presentations. Comparative data on the severity of these infections in hospitalized adults are limited, particularly across phases of the COVID-19 pandemic. **Objectives:** The objectives of this study are to compare the risk of severe outcomes among hospitalized patients with COVID-19, influenza, or RSV and to evaluate the role of vaccination and demographic subgroups using recent, real-world data. **Design:** This is a retrospective cohort study. Setting: Eight hospitals within the Corewell Health system in Michigan, USA, were studied. **Participants:** The participants included adults aged ≥ 18 years hospitalized between 1 January 2021 and 20 July 2024 with a principal diagnosis of COVID-19, influenza, or RSV. **Main Outcomes and Measures:** The primary outcome was a composite of ICU admission, mechanical ventilation, or in-hospital death. Multivariable Cox proportional hazard models were used to estimate adjusted hazard ratios (aHRs), with subgroup analyses in terms of vaccination status, age group, and time period. **Results:** Among 27,885 hospitalized patients (90.5% COVID-19, 7.2% influenza, 2.3% RSV), COVID-19 was associated with a higher risk of severe outcomes compared to influenza (aHR 1.30, 95% CI: 1.11–1.54). RSV showed no significant difference from influenza. Across all infection groups, older age (≥65 years), high comorbidity burden, and immunocompromised status were associated with an increased risk of severe outcomes. Recent COVID-19 vaccination was protective, particularly among older adults. Differences in severity were more pronounced in the pre-March 2022 period. **Conclusions:** Using one of the most recent large-scale datasets, this study is among the first to directly compare the severity of COVID-19, influenza, and RSV in hospitalized adults. COVID-19 continues to pose a higher risk of severe illness compared to the other viral infections. The findings underscore the importance of up-to-date vaccination and focused clinical strategies for older and high-risk individuals. This study offers timely evidence to guide future respiratory virus response strategies across hospital settings.

## 1. Introduction

Viral respiratory illnesses are a significant public health concern in the United States, with influenza, respiratory syncytial virus (RSV), and COVID-19 being among the most common culprits. Each year, approximately 8% of the U.S. population is affected by influenza, while RSV remains the leading cause of outpatient visits in children under two years old [1,2]. Since its emergence in Wuhan, China, in 2019, COVID-19 has resulted in over 1.2 million deaths in the U.S. [3]. In recent months, a rise in RSV- and influenza-related emergency visits and hospitalizations has underscored that, beyond SARS-CoV-2, other respiratory viruses also contribute to severe outcomes [4,5].

These viruses primarily spread through respiratory droplets and direct contact with secretions, though some evidence suggests airborne transmission [6]. Common symptoms include fever, myalgias, cough, shortness of breath, and nasal congestion [7,8]. While many cases are self-limiting, severe infections can progress to viral pneumonia, sepsis, and multi-organ failure [9,10]. Some studies indicate similar rates of severe outcomes across these viruses, while others highlight some differences [1,2,11]. Understanding these variations and identifying vulnerable populations are essential for guiding prevention and treatment strategies. This study aims to clarify differences in clinical outcomes among patients with COVID-19, RSV, and influenza to inform more effective therapeutic and public health interventions.

However, few studies have concurrently evaluated these three infections using a uniform analytic approach with updated real-world data. Our study addresses this gap by comparing the severity of COVID-19, influenza, and RSV across different pandemic phases and vaccination statuses in a large, diverse hospital cohort.

## 2. Materials and Methods

### 2.1. Study Design

This observational, retrospective, multicenter study examined emergency department (ED) patients hospitalized with acute RSV, influenza, or COVID-19. The study was conducted across eight hospitals in southeastern Michigan, representing a diverse healthcare landscape that includes small community hospitals and a large academic tertiary care center.

### 2.2. Selection of Participants

Patients were included if they were 18 years or older and presented to the ED at one of Corewell Health East’s eight hospitals between 1 January, 2021 and 20 July, 2024, with a principal diagnosis of COVID-19, RSV, or influenza. This study was approved by the Corewell Health Institutional Review Board (IRB), and due to its retrospective nature, a waiver for written informed consent was granted. This study was conducted in accordance with institutional ethical standards for human subject research and the principles outlined in the Helsinki Declaration.

### 2.3. Data Source

All data were extracted from the electronic health record (EHR) system (Epic, Verona, WI, USA), including demographic, clinical, laboratory, and outcome variables. These variables encompassed age, sex, ethnicity, past medical history, vaccination status, initial vital signs, in-hospital therapies, and key outcomes such as ICU admission, need for mechanical ventilation, mortality, and hospital length of stay. Comorbidities were assessed using the Agency for Healthcare Research and Quality (AHRQ) Elixhauser Comorbidity Index. Immunocompromised status was determined based on International Classification of Diseases, 10th Revision (ICD-10) codes, following the AHRQ definition, which includes autoimmune diseases, organ transplants, nutritional deficiencies, specific genetic conditions, certain chronic diseases, and human immunodeficiency virus (HIV) infection. Vaccination status for SARS-CoV-2 and influenza was verified through the institutional EHR, which is linked to the Michigan Care Improvement Registry (MCIR). This integration ensured accurate vaccination data, including records for patients immunized outside the Corewell Health System. The MCIR maintains comprehensive SARS-CoV-2 vaccination records for individuals immunized in Michigan, including vaccine type and administration date.

### 2.4. Definitions

Composite severe infection was defined as mechanical ventilation, ICU admission, or mortality at any point during hospitalization. Time to severe infection was calculated from the time of hospital arrival to the occurrence of any of the composite events.

Vaccination status was classified based on timing relative to hospital arrival. COVID-19 vaccine status was defined as recent (received within 1 year), old (received more than 1 year ago), or never (no prior COVID-19 vaccination). Influenza vaccine status was defined as yes (received within the past year) or no (no record of influenza vaccination in the past year). This classification was used to account for potential waning immunity over time, consistent with the prior literature showing reduced vaccine effectiveness beyond 12 months [12,13]. RSV vaccination status was not included in this analysis due to its limited availability during the study period and inconsistent documentation within the electronic health record system.

The time period was stratified as pre-March 2022 and post-March 2022. March 2022 was selected as the cutoff to distinguish between early and later phases of the pandemic, coinciding with the widespread circulation of the Omicron variant and expanded vaccine coverage. This division reflects shifts in viral characteristics, population immunity, and clinical management, as supported by trends in case volume and variant distribution shown in Supplementary Figure S1 [13,14].

### 2.5. Outcomes

The primary objective of this study was to compare the frequency of composite severe outcomes among patients hospitalized in the emergency department with RSV, influenza, or COVID-19. Severe infection was defined as a composite outcome consisting of intensive care unit (ICU) admission, mechanical ventilation, or in-hospital death.

The secondary objective included evaluating the association of vaccination status, older age, and time period with severe clinical outcomes. For vaccination analysis, this assessment was limited to patients with influenza or COVID-19.

### 2.6. Statistical Analysis

Descriptive statistics were used to summarize patient characteristics across infection groups. Continuous variables were reported as means with standard deviations (SDs) and compared using ANOVA tests. Categorical variables were summarized as counts and percentages and compared using Chi-square tests.

To assess the time to severe infection, we employed Kaplan–Meier survival analysis stratified by infection type. Multivariable Cox proportional hazard regression models were used to examine the association between infection type and the risk of severe infection, a composite outcome of ICU admission, mechanical ventilation, or in-hospital death. The covariates included in the models were age, sex, race, Elixhauser Comorbidity Index, vaccination status (COVID-19 and influenza), immunocompromised status, and time period.

To further explore effect modification, subgroup analyses were performed. We used Cox regression models stratified by age group (18–49, 50–64, 65) and by time period (pre-March 2022 vs. post-March 2022). All models were adjusted for the same covariates, excluding the stratification variable. Descriptive characteristics for each subgroup are summarized in Supplementary Tables S1 and S2, respectively.

Adjusted hazard ratios were reported with corresponding 95% confidence intervals (CIs) and *p*-values for the Cox proportional hazard regression analysis. All statistical tests were two-sided, and statistical significance was determined using a *p*-value threshold of less than 0.05. The analysis was conducted using R-4.3.1, provided by the R Foundation for Statistical Computing.

## 3. Results

**Figure 1:**
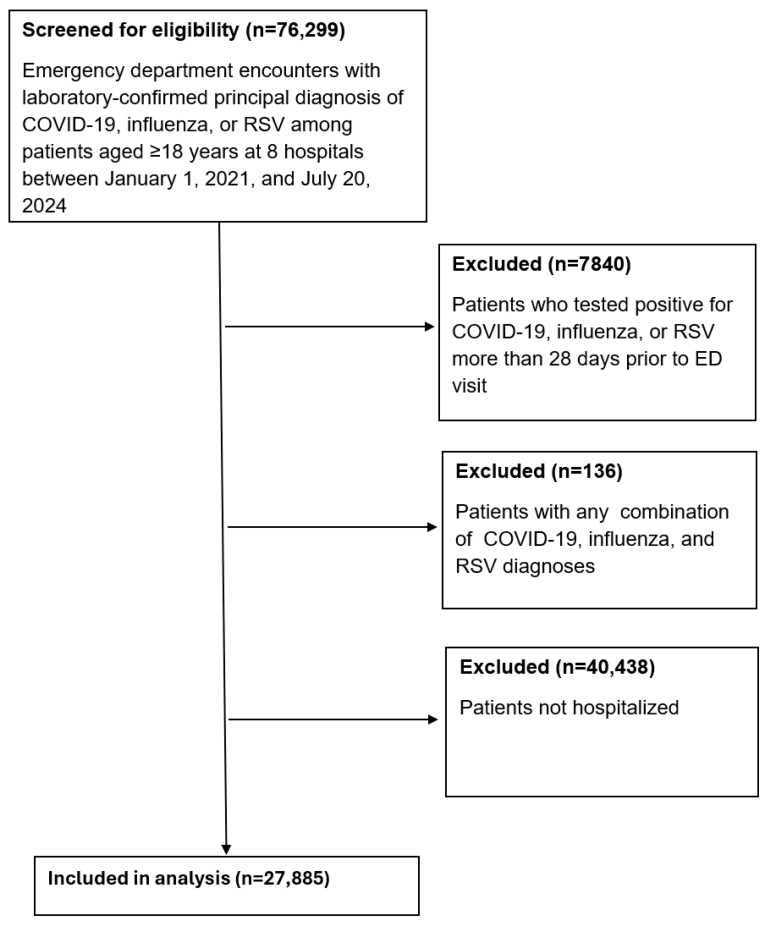
Flow chart of inclusion and exclusion criteria. Flow chart demonstrating total number of encounters screened for eligibility, number of excluded encounters, and final number of encounters included in analysis.

From 1 January 2021 to 20 July 2024, 76,299 encounters were screened for eligibility (Figure 1). A total of 40,438 patients who were not hospitalized following their ED visit were excluded. Additionally, 136 patients with any combination of COVID-19, RSV, and influenza were excluded to ensure mutually exclusive groups. Another 7840 patients were excluded because they had tested positive for one of the three viruses more than 28 days prior to the ED encounter, which was considered non-acute. After applying these criteria, 27,885 patients were included in the final analysis cohort. Among them, 2013 (7.2%) had influenza, 635 (2.3%) had RSV, and 25,237 (90.5%) had COVID-19. RSV and influenza patients had lower rates of ED utilization compared to COVID-19 patients, although the pattern varied by age and comorbidity profile. The mean age was 65.2 years (SD 17.7), with 55.5% of patients aged 65 or older. Overall, 52.9% of the patients were female. COVID-19 patients were slightly younger on average and more likely to be unvaccinated against COVID-19. RSV patients had the highest comorbidity burden, with 50.1% having an Elixhauser Comorbidity Index ≥ 5 (Table 1).

**Figure 2:**
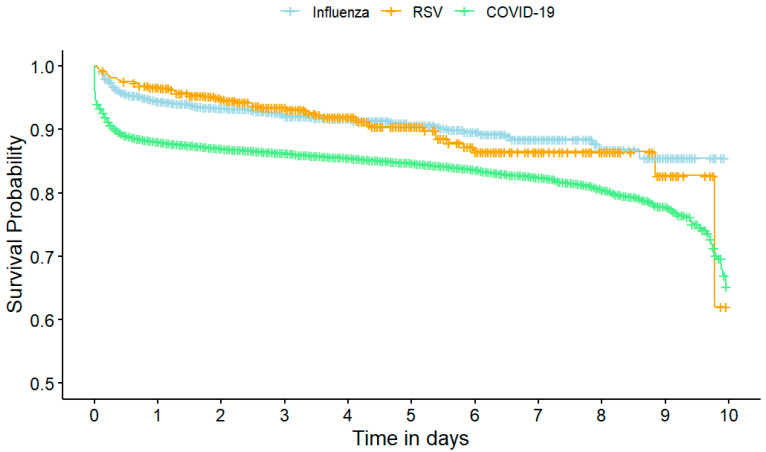
A Kaplan–Meier plot of severe outcome. The x-axis indicates the length of hospital stay in days. The y-axis represents the probability of survival without developing severe infection. Patients with hospital stays longer than 10 days are censored.

Overall, 13.7% (n = 3819) of patients developed severe disease. The rate of severe disease was the lowest among influenza patients (8.1%), followed by RSV (9.1%), and the highest among COVID-19 patients (14.3%) (Table 1). Kaplan–Meier analysis revealed that patients with COVID-19 had the lowest survival probability without severe disease compared to influenza and RSV (Figure 2).

**Figure 3:**
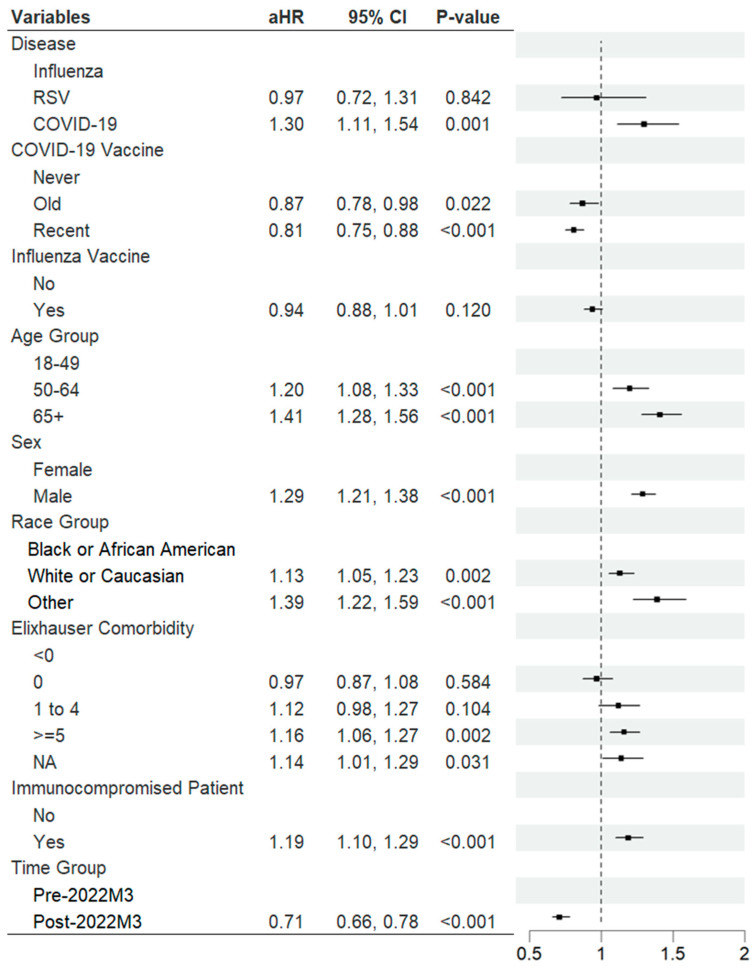
Multivariable Cox proportional hazard regression for risk of severe outcome. Abbreviations: aHR = adjusted hazard ratio; CI = confidence interval. Model adjusted for age, sex, race, comorbidities, vaccination status, immunocompromised status, and time period.

In the Cox proportional hazard regression adjusted for age, sex, race, comorbidities, immunocompromised status, vaccination status, and time period, infection type was significantly associated with the risk of severe infection (Figure 3). Compared to influenza, COVID-19 was associated with a significantly higher risk of severe disease (adjusted hazard ratio (aHR) 1.30, 95% CI: 1.11–1.54), while RSV showed a modest and not statistically significant difference.

Analyses stratified by age showed that the association between disease type and severe outcome varied by age group (Supplementary Figure S2, Supplementary Table S1). In the 50–64 and ≥ 65 age groups, COVID-19 was significantly associated with an increased risk of severe disease compared to influenza (aHR 1.50 (95% CI: 1.05–2.15) and 1.29 (95% CI: 1.05–1.58), respectively). In patients aged 18–49, the difference was not statistically significant. Recent COVID-19 vaccination was associated with a reduced risk of severe disease in the older age groups (Supplementary Table S2).

We further stratified the analysis by pre- vs. post-March 2022 (Supplementary Figure S3, Supplementary Table S3). The risk of severe COVID-19 was significantly higher than that for influenza during the pre-March 2022 period (aHR 3.13, 95% CI: 1.01–9.71), while the association was attenuated in the post-March 2022 period (aHR 1.27, 95% CI: 1.07–1.50). Recent COVID-19 vaccination remained protective in the earlier period but not in the later one. The effects of age, comorbidities, and immunocompromised status on severe infection risk remained consistent across time periods (Supplementary Table S4).

## 4. Discussion

In this large retrospective cohort study of over 27,000 hospitalized patients with respiratory infections, we found that COVID-19 was associated with a significantly higher risk of severe clinical outcomes compared to influenza and RSV, even after adjusting for key demographic and clinical factors. Our findings provide updated, real-world evidence on the comparative severity of these viral infections during a dynamic period of the pandemic and shifting viral epidemiology. To our knowledge, this is one of the first large-scale studies directly comparing the clinical severity of influenza, RSV, and COVID-19 in a single cohort using the most recent available data.

Consistent with prior research, COVID-19 was linked to a greater risk of ICU admission, mechanical ventilation, or in-hospital death [15,16]. While RSV patients demonstrated high comorbidity burden, their adjusted risk of severe outcomes was not significantly different from that for influenza patients. The markedly higher risk observed in COVID-19 patients reinforces the ongoing clinical burden posed by SARS-CoV-2 despite increasing population immunity and evolving viral strains [12]. Surie et al. (2023) [5] reported the greater severity of RSV compared to both influenza and COVID-19 among hospitalized adults aged ≥ 60 years. In contrast, our study found no significant difference in severity between RSV and influenza, even within older subgroups. A key distinction is that our analysis employed Kaplan–Meier survival curves and Cox proportional hazard regression, allowing for time-to-event modeling and adjustment for censoring, whereas Surie et al. used static risk comparisons. Furthermore, our models controlled for comorbidity burden, COVID-19 and influenza vaccination status, and immunocompromised status—factors that were not included in the Surie study. These differences in analytic approach and adjustment likely contribute to the contrasting findings.

Our subgroup analyses revealed important effect modifications by age and time period. Among adults aged 50 years and older, COVID-19 remained significantly associated with a greater risk of severe infection compared to influenza. In contrast, among younger adults aged 18–49, the difference in severity across infection types was not statistically significant. These age-stratified findings highlight the particular vulnerability of older populations to COVID-19-related complications [17].

The association between pathogen type and severe outcome was also temporally dependent. During the early phase of the pandemic (pre-March 2022), COVID-19 was associated with an over threefold higher risk of severe infection compared to influenza, whereas the difference was less pronounced in the post-March 2022 period. This attenuation may reflect improvements in clinical management, broader vaccine coverage, and a possibly reduced virulence of newer SARS-CoV-2 variants [14,18].

Vaccination status played a critical role in mitigating severe outcomes. Recent COVID-19 vaccination was associated with a lower risk of severe disease, particularly among older adults. These findings align with prior studies demonstrating the effectiveness of vaccination in reducing hospitalization severity, even as vaccine protection against disease may wane over time [13,19]. Interestingly, influenza vaccination did not appear to significantly impact the severity of illness in our adjusted models, suggesting differences in vaccine-mediated protection between these viruses [20].

Also, comorbidity burden—as measured by the Elixhauser Comorbidity Index—was significantly associated with severe outcome risk. Patients with higher comorbidity scores, particularly those with an index of 5 or more, exhibited increased hazards of ICU admission, mechanical ventilation, or death. Notably, the association between comorbidity burden and severe outcomes was the strongest among patients aged 65 and older, as well as during the post-March 2022 period. These observations underscore the compounding vulnerability of older adults with complex medical conditions and the need for comprehensive risk stratification in clinical care, as well as the importance of focused interventions in this population.

Additionally, we observed that immunocompromised patients had a consistently higher risk of severe infection across all models. This association was especially evident in the post-March 2022 period and among adults aged 50–64, highlighting the heightened vulnerability of immunocompromised individuals despite broader population-level improvements in disease outcomes. These findings support targeted preventive strategies and close monitoring for this high-risk subgroup.

As the landscape of respiratory viruses continues to evolve, ongoing surveillance and real-time data evaluation will be crucial to adapt vaccination strategies and prioritize high-risk populations.

## 5. Limitations

This study has several limitations that should be considered when interpreting the findings. First, as a retrospective analysis based on electronic health record (EHR) data, there is potential for the misclassification of infection type, vaccination status, and clinical outcomes due to documentation errors or incomplete records. Second, although we adjusted for numerous clinical and demographic covariates, residual confounding may still exist. Important variables such as treatment details (e.g., antiviral or immunomodulatory use) and socioeconomic factors were not consistently available and could influence outcomes. Third, the definition of severe disease was based on a composite outcome (ICU admission, mechanical ventilation, or in-hospital death), which, while clinically meaningful, may not capture the full spectrum of disease severity or differentiate among severity levels. Finally, this study was conducted within a single region, which may limit the generalizability of the findings to other populations or settings with different demographics, healthcare infrastructure, or pandemic dynamics.

## 6. Conclusions

In this large real-world cohort study comparing influenza, RSV, and COVID-19 among hospitalized patients, COVID-19 was consistently associated with the highest risk of severe clinical outcomes, including ICU admission, mechanical ventilation, and in-hospital death. These findings held true across multiple analytic approaches, with particularly elevated risks among older adults, individuals with high comorbidity burden, and immunocompromised patients. Temporal analyses revealed that while the relative severity of COVID-19 decreased over time, it remained higher than that of other respiratory infections, underscoring its continued impact on vulnerable populations. Vaccination, especially recent COVID-19 vaccination, was associated with a lower risk of severe outcomes, reinforcing its public health importance. These results support the need for continued vigilance, preventive strategies, and resource planning for COVID-19 and other respiratory diseases, particularly during periods of high circulation. Tailored interventions for high-risk groups remain essential to reduce morbidity and mortality in future respiratory infection surges.

## Figures and Tables

**Table 1:** Patient characteristics by disease.

Variables *	All	Influenza	RSV	COVID-19	*p*-Value
n	27,885	2013 (7.2%)	635 (2.3%)	25,237 (90.5%)	
Severe Outcome					<0.001 ^1^
No	24,066 (86.3%)	1850 (91.9%)	577 (90.9%)	21,639 (85.7%)	
Yes	3819 (13.7%)	163 (8.1%)	58 (9.1%)	3598 (14.3%)	
Age					<0.001 ^2^
Mean (SD)	65.2 (17.7)	64.7 (18.0)	69.4 (16.6)	65.1 (17.7)	
Age Group					<0.001 ^1^
18–49	5378 (19.3%)	396 (19.7%)	74 (11.7%)	4908 (19.4%)	
50–64	7021 (25.2%)	474 (23.5%)	146 (23.0%)	6401 (25.4%)	
65+	15,486 (55.5%)	1143 (56.8%)	415 (65.4%)	13,928 (55.2%)	
Sex					<0.001 ^1^
Female	14,757 (52.9%)	1162 (57.7%)	431 (67.9%)	13,164 (52.2%)	
Male	13,128 (47.1%)	851 (42.3%)	204 (32.1%)	12,073 (47.8%)	
Race					<0.001 ^1^
Black or African American	7145 (25.6%)	612 (30.4%)	147 (23.1%)	6386 (25.3%)	
White or Caucasian	18,994 (68.1%)	1261 (62.6%)	453 (71.3%)	17,280 (68.5%)	
Other	1746 (6.3%)	140 (7.0%)	35 (5.5%)	1571 (6.2%)	
Elixhauser Comorbidity Index					<0.001 ^1^
<0	5709 (20.5%)	454 (22.6%)	127 (20.0%)	5128 (20.3%)	
0	5937 (21.3%)	341 (16.9%)	83 (13.1%)	5513 (21.8%)	
1–4	2483 (8.9%)	217 (10.8%)	60 (9.4%)	2206 (8.7%)	
>=5	11,072 (39.7%)	861 (42.8%)	318 (50.1%)	9893 (39.2%)	
N/A	2684 (9.6%)	140 (7.0%)	47 (7.4%)	2497 (9.9%)	
COVID-19 Vaccine					<0.001 ^1^
Never	13,203 (47.3%)	547 (27.2%)	141 (22.2%)	12,515 (49.6%)	
Old	4995 (17.9%)	818 (40.6%)	225 (35.4%)	3952 (15.7%)	
Recent	9687 (34.7%)	648 (32.2%)	269 (42.4%)	8770 (34.8%)	
Influenza Vaccine					<0.001 ^1^
No	18,147 (65.1%)	1336 (66.4%)	353 (55.6%)	16,458 (65.2%)	
Yes	9738 (34.9%)	677 (33.6%)	282 (44.4%)	8779 (34.8%)	
Immunocompromised Patient					<0.001 ^1^
No	22,617 (81.1%)	1685 (83.7%)	492 (77.5%)	20,440 (81.0%)	
Yes	5268 (18.9%)	328 (16.3%)	143 (22.5%)	4797 (19.0%)	
ICU Admission					<0.001 ^1^
No	24,546 (88.0%)	1863 (92.5%)	580 (91.3%)	22,103 (87.6%)	
Yes	3339 (12.0%)	150 (7.5%)	55 (8.7%)	3134 (12.4%)	
Mechanical ventilation					<0.001 ^1^
No	26,108 (93.6%)	1958 (97.3%)	607 (95.6%)	23,543 (93.3%)	
Yes	1777 (6.4%)	55 (2.7%)	28 (4.4%)	1694 (6.7%)	
In-Hospital Death					<0.001 ^1^
No	26,276 (94.2%)	1982 (98.5%)	619 (97.5%)	23,675 (93.8%)	
Yes	1609 (5.8%)	31 (1.5%)	16 (2.5%)	1562 (6.2%)	
Time Group					<0.001 ^1^
Pre-2022 March	14,897 (53.4%)	61 (3.0%)	141 (22.2%)	14,695 (58.2%)	
Post-2022 March	12,988 (46.6%)	1952 (97.0%)	494 (77.8%)	10,542 (41.8%)	
Time to Severe Infection					<0.001 ^2^
Mean (SD)	5.1 (5.4)	4.0 (3.5)	5.1 (4.2)	5.2 (5.5)	

* For continuous variables, means (standard deviations) are presented. For categorical variables, frequencies (percentage) are presented. COVID-19 Vaccine: “Old” indicates vaccination more than one year ago; “Recent” indicates vaccination within the past year. Influenza Vaccine: “Yes” indicates vaccination within the past year. ^1^ Chi-square test. ^2^ ANOVA test.

## Data Availability

The data that support the findings of this study are available via a data access agreement. Please contact the corresponding author (AB) regarding this request.

## Supplementary Materials

The following supporting information can be downloaded at: https://www.mdpi.com/article/10.3390/jcm14144894/s1, Supplementary Figure S1. ED encounters among infections groups. Supplementary Figure S2. Multivariable Cox Proportional Hazards Regressions for Risk of Severe Infection Stratified by Age Group. Supplementary Figure S3. Multivariable Cox Proportional Hazards Regressions for Risk of Severe Infection Stratified by Time Period. Supplementary Table S1. Patient characteristics by age group. Supplementary Table S2. Multivariable Cox proportional hazards regressions for risk of severe infection stratified by age group. Supplementary Table S3. Patient characteristics by time period. Supplementary Table S4. Multivariable Cox proportional hazards regressions for risk of severe infection stratified by time period.
