# Supplementary material for: Comparative Analysis of Severe Clinical Outcomes in Hospitalized Patients with RSV, Influenza, and COVID-19 Across Early and Late COVID-19 Pandemic Phases (2021–2024)
Source: J Clin Med. 2025 Jul 10;14(14):4894. doi: 10.3390/jcm14144894 (PMC12295860; doi:10.3390/jcm14144894)
Supplement: Supplementary file 1 [file jcm-14-04894-s001.zip › jcm-3706063-supplementary.pdf]

Supplementary Figure S1. ED encounters among infections groups

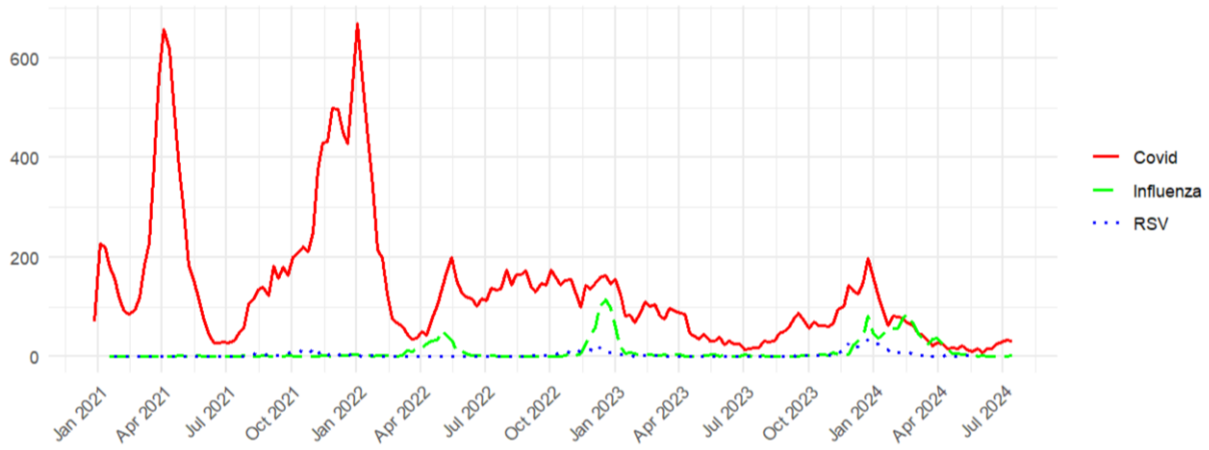

Supplementary Figure S2. Multivariable Cox Proportional Hazards Regressions for Risk of Severe Infection Stratified by Age Group

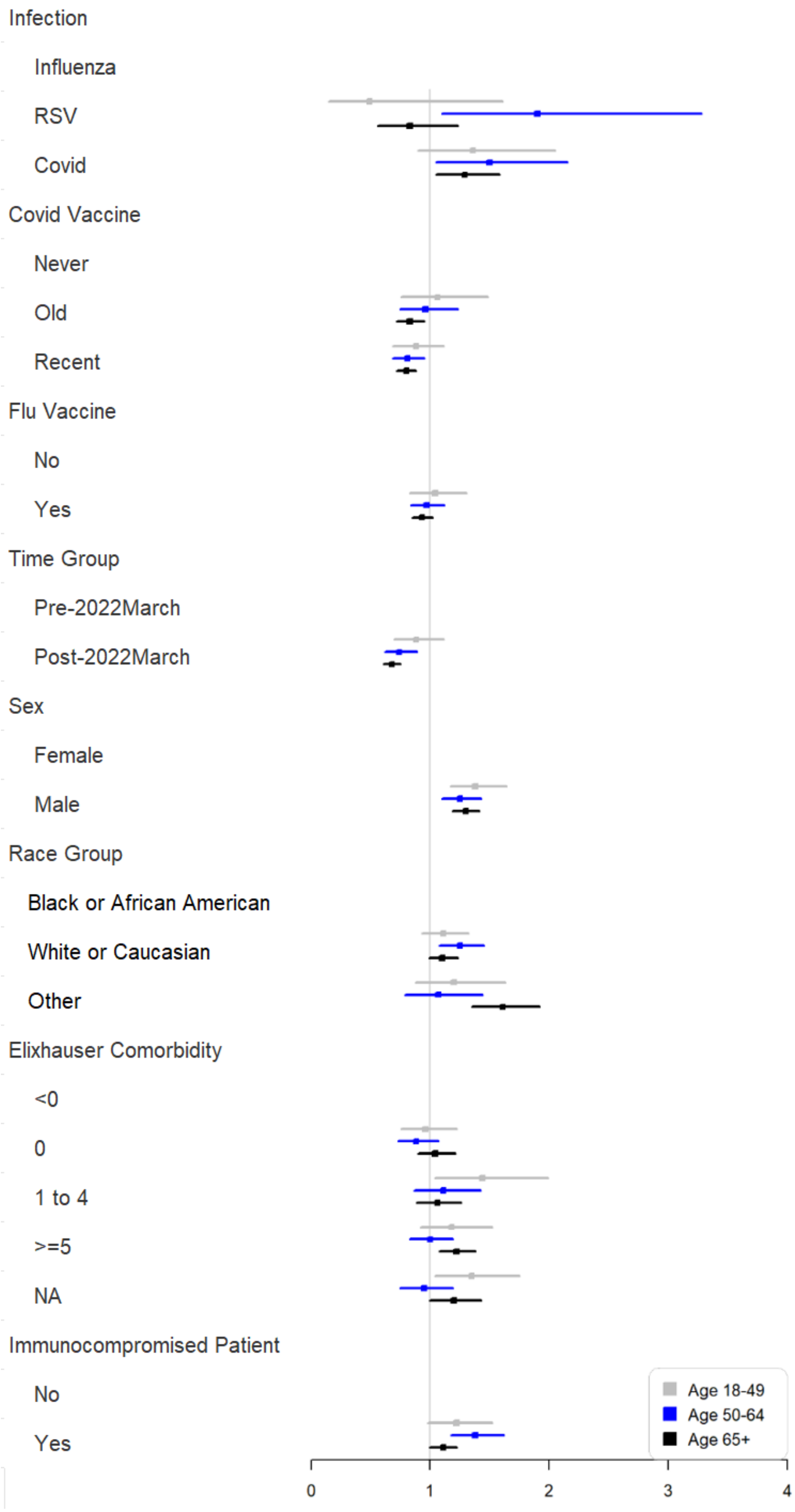

Each panel shows the results of a multivariable Cox proportional hazards regression model for a specific age group. Hazard ratios (HRs) and 95% confidence intervals (CIs) are shown. Models are adjusted for sex, race, comorbidities, vaccination status, immunocompromised status and time period.

Supplementary Figure S3. Multivariable Cox Proportional Hazards Regressions for Risk of Severe Infection Stratified by Time Period

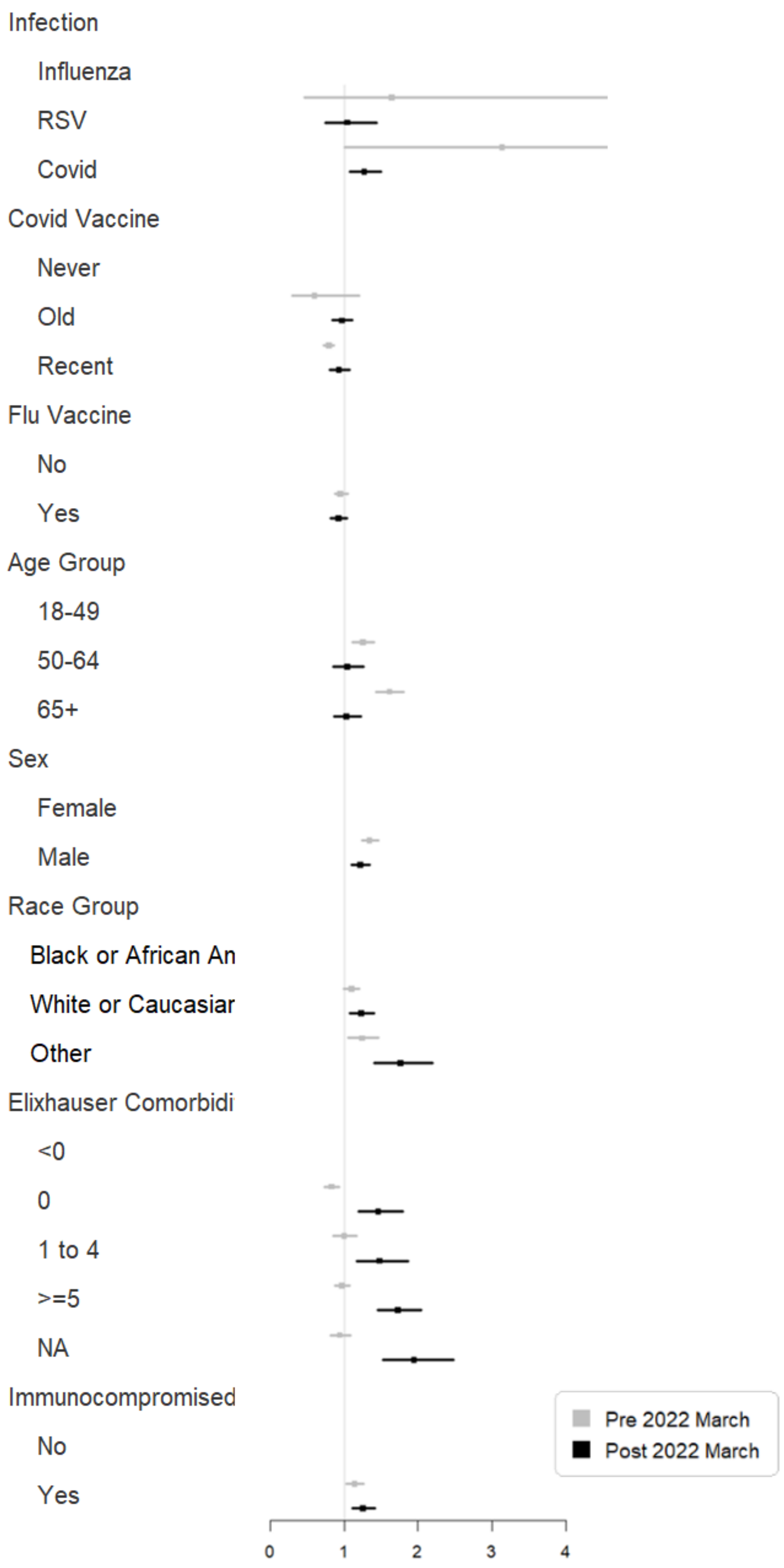

Each panel shows the results of a multivariable Cox proportional hazards regression model for a specific time period. Hazard ratios (HRs) and 95% confidence intervals (CIs) are shown. Models are adjusted for age, sex, race, comorbidities, vaccination status, and immunocompromised status.

Supplementary Table S1. Patient characteristics by age group

Variables*	All	18-49	50-64	65+	P value
n	27885	5378 (19.3%)	7021 (25.2%)	15486 (55.5%)	
Infection					< 0.001[1]
Influenza	2013 (7.2%)	396 (7.4%)	474 (6.8%)	1143 (7.4%)	
RSV	635 (2.3%)	74 (1.4%)	146 (2.1%)	415 (2.7%)	
Covid	25237 (90.5%)	4908 (91.3%)	6401 (91.2%)	13928 (89.9%)	
Severe Infection					< 0.001[1]
No	24066 (86.3%)	4801 (89.3%)	6089 (86.7%)	13176 (85.1%)	
Yes	3819 (13.7%)	577 (10.7%)	932 (13.3%)	2310 (14.9%)	
Age					< 0.001[2]
Mean (SD)	65.12 (17.7)	37.5 (8.5)	57.7 (4.3)	78.2 (8.52)	
Sex					< 0.001[1]
Female	14757 (52.9%)	2770 (51.5%)	3557 (50.7%)	8430 (54.4%)	
Male	13128 (47.1%)	2608 (48.5%)	3464 (49.3%)	7056 (45.6%)	
Race Group					< 0.001[1]
Black or African American	7145 (25.6%)	2095 (39.0%)	2117 (30.2%)	2933 (18.9%)	
White or Caucasian	18994 (68.1%)	2847 (52.9%)	4469 (63.7%)	11678 (75.4%)	
Other	1746 (6.3%)	436 (8.1%)	435 (6.2%)	875 (5.7%)	
Elixhauser Comorbidity Index					< 0.001[1]
<0	5709 (20.5%)	1336 (24.8%)	1758 (25.0%)	2615 (16.9%)	
0	5937 (21.3%)	1613 (30.0%)	1631 (23.2%)	2693 (17.4%)	
1 to 4	2483 (8.9%)	408 (7.6%)	627 (8.9%)	1448 (9.4%)	
>=5	11072 (39.7%)	1215 (22.6%)	2207 (31.4%)	7650 (49.4%)	
N/A	2684 (9.6%)	806 (15.0%)	798 (11.4%)	1080 (7.0%)	
Covid Vaccine					< 0.001[1]
Never	13203 (47.3%)	3850 (71.6%)	3971 (56.6%)	5382 (34.8%)	
Old	4995 (17.9%)	563 (10.5%)	1023 (14.6%)	3409 (22.0%)	
Recent	9687 (34.7%)	965 (17.9%)	2027 (28.9%)	6695 (43.2%)	
Flu Vaccine					< 0.001[1]
No	18147 (65.1%)	4420 (82.2%)	4917 (70.0%)	8810 (56.9%)	
Yes	9738 (34.9%)	958 (17.8%)	2104 (30.0%)	6676 (43.1%)	
Immunocompromised Patient					< 0.001[1]
No	22617 (81.1%)	4535 (84.3%)	5590 (79.6%)	12492 (80.7%)	
Yes	5268 (18.9%)	843 (15.7%)	1431 (20.4%)	2994 (19.3%)	
Time Group					< 0.001[1]
Pre-2022M3	14897 (53.4%)	3697 (68.7%)	4430 (63.1%)	6770 (43.7%)	
Post-2022M3	12988 (46.6%)	1681 (31.3%)	2591 (36.9%)	8716 (56.3%)	
Time to Severe Infection					< 0.001[2]
Mean (SD)	5.1 (5.4)	4.04 (4.1)	5.0 (5.9)	5.6 (5.4)	

*For continuous variables, mean (standard deviation) were presented. For categorical variables, frequencies (percentage) were presented. Covid Vaccine: "Old" indicates vaccination more than one year ago; "Recent" indicates vaccination within the past year. Flu Vaccine: "Yes" indicates vaccination within the past year.

[1]Chi-square test

[2]ANOVA-test

Supplementary Table S2. Multivariable Cox proportional hazards regressions for risk of severe infection stratified by age group

	Age 18-49			Age 50-64			Age 65+		
**Variables**	**aHR**	**95% CI**	**P-value**	**aHR**	**95% CI**	**P-value**	**aHR**	**95% CI**	**P-value**
Infection									
Influenza									
RSV	0.49	0.15, 1.61	0.239	1.90	1.10, 3.28	0.021	0.83	0.56, 1.23	0.358
Covid	1.36	0.90, 2.05	0.140	1.50	1.05, 2.15	0.027	1.29	1.05, 1.58	0.016
Covid Vaccine									
Never									
Old	1.06	0.76, 1.48	0.734	0.96	0.75, 1.23	0.744	0.83	0.72, 0.95	0.009
Recent	0.88	0.69, 1.11	0.289	0.81	0.69, 0.95	0.009	0.80	0.72, 0.88	<0.001
Flu Vaccine									
No									
Yes	1.04	0.83, 1.30	0.738	0.97	0.84, 1.12	0.667	0.93	0.85, 1.02	0.126
Time Group									
Pre-2022March									
Post-2022March	0.88	0.70, 1.11	0.281	0.74	0.62, 0.89	0.001	0.68	0.61, 0.75	<0.001
Sex									
Female									
Male	1.38	1.17, 1.64	<0.001	1.25	1.10, 1.43	<0.001	1.30	1.19, 1.41	<0.001
Race Group									
Black or African American									
White or Caucasian	1.11	0.93, 1.32	0.265	1.25	1.08, 1.45	0.003	1.10	0.99, 1.23	0.074
Other	1.20	0.88, 1.63	0.258	1.07	0.79, 1.44	0.673	1.61	1.35, 1.92	<0.001
Elixhauser Comorbidity									
<0									
0	0.96	0.76, 1.22	0.738	0.88	0.73, 1.07	0.208	1.04	0.90, 1.21	0.575
1 to 4	1.44	1.04, 1.99	0.029	1.11	0.87, 1.42	0.413	1.06	0.89, 1.26	0.535
>=5	1.18	0.92, 1.52	0.185	1.00	0.83, 1.19	0.964	1.22	1.08, 1.38	0.001
NA	1.35	1.04, 1.75	0.025	0.95	0.75, 1.19	0.631	1.20	1.00, 1.43	0.045
Immunocompromised Patient									
No									

Yes	1.22	0.98, 1.52	0.080	1.38	1.18, 1.62	<0.001	1.11	1.00, 1.22	0.052

Abbreviations: aHR = adjusted hazard ratio, CI = confidence interval.
Model adjusted for sex, race, comorbidities, vaccination status, immunocompromised status and time period.

Supplementary Table S3. Patient characteristics by time period

Variables*	All	Pre 2022 March	Post 2022 March	P value
n	27885	14897(53.4%)	12988(46.6%)	
Infection				< 0.001[1]
Influenza	2013 (7.2%)	61 (0.4%)	1952 (15.0%)	
RSV	635 (2.3%)	141 (0.9%)	494 (3.8%)	
Covid	25237 (90.5%)	14695 (98.6%)	10542 (81.2%)	
Severe Infection				< 0.001[1]
No	24066 (86.3%)	12479 (83.8%)	11587 (89.2%)	
Yes	3819 (13.7%)	2418 (16.2%)	1401 (10.8%)	
Age				< 0.001[2]
Mean (SD)	65.177 (17.743)	61.474 (17.423)	69.424 (17.146)	
Age Group				< 0.001[1]
18-49	5378 (19.3%)	3697 (24.8%)	1681 (12.9%)	
50-64	7021 (25.2%)	4430 (29.7%)	2591 (19.9%)	
65+	15486 (55.5%)	6770 (45.4%)	8716 (67.1%)	
Sex				< 0.001[1]
Female	14757 (52.9%)	7563 (50.8%)	7194 (55.4%)	
Male	13128 (47.1%)	7334 (49.2%)	5794 (44.6%)	
Race Group				< 0.001[1]
Black or African American	7145 (25.6%)	3975 (26.7%)	3170 (24.4%)	
White or Caucasian	18994 (68.1%)	9925 (66.6%)	9069 (69.8%)	
Other	1746 (6.3%)	997 (6.7%)	749 (5.8%)	
Elixhauser_Comorbidity				< 0.001[1]
<0	5709 (20.5%)	3245 (21.8%)	2464 (19.0%)	
0	5937 (21.3%)	3757 (25.2%)	2180 (16.8%)	
1 to 4	2483 (8.9%)	1211 (8.1%)	1272 (9.8%)	
>=5	11072 (39.7%)	4832 (32.4%)	6240 (48.0%)	
N/A	2684 (9.6%)	1852 (12.4%)	832 (6.4%)	
Covid Vaccine				< 0.001[1]
Never	13203 (47.3%)	10175 (68.3%)	3028 (23.3%)	
Old	4995 (17.9%)	72 (0.5%)	4923 (37.9%)	
Recent	9687 (34.7%)	4650 (31.2%)	5037 (38.8%)	
Flu Vaccine				< 0.001[1]
No	18147 (65.1%)	10564 (70.9%)	7583 (58.4%)	
Yes	9738 (34.9%)	4333 (29.1%)	5405 (41.6%)	
Immunocompromised Patient				< 0.001[1]
No	22617 (81.1%)	12274 (82.4%)	10343 (79.6%)	
Yes	5268 (18.9%)	2623 (17.6%)	2645 (20.4%)	
Time to Severe Infection				< 0.001[2]
Mean (SD)	5.136 (5.372)	5.473 (5.804)	4.751 (4.801)	

*For continuous variables, mean (standard deviation) were presented. For categorical variables, frequencies (percentage) were presented. Covid Vaccine: "Old" indicates vaccination more than one year ago; "Recent" indicates vaccination within the past year. Flu Vaccine: "Yes" indicates vaccination within the past year.

[1]Chi-square test

[2]ANOVA-test

Supplementary Table S4. Multivariable Cox proportional hazards regressions for risk of severe infection stratified by time period

	Pre 2022 March			Post 2022 March		
**Variables**	**aHR**	**95% CI**	**P-value**	**aHR**	**95% CI**	**P-value**
Infection						
Influenza						
RSV	1.64	0.46, 5.89	0.446	1.04	0.75, 1.44	0.830
Covid	3.13	1.01, 9.71	0.049	1.27	1.07, 1.50	0.005
Covid Vaccine						
Never						
Old	0.60	0.30, 1.20	0.148	0.97	0.84, 1.11	0.636
Recent	0.79	0.72, 0.86	<0.001	0.93	0.80, 1.08	0.354
Flu Vaccine						
No						
Yes	0.95	0.87, 1.05	0.317	0.92	0.82, 1.04	0.174
Age Group						
18-49						
50-64	1.25	1.11, 1.41	<0.001	1.04	0.85, 1.27	0.709
65+	1.61	1.43, 1.81	<0.001	1.03	0.86, 1.23	0.756
Sex						
Female						
Male	1.34	1.24, 1.46	<0.001	1.22	1.10, 1.35	<0.001
Race Group						
Black or African American						
White or Caucasian	1.10	0.99, 1.21	0.065	1.23	1.07, 1.40	0.003
Other	1.24	1.05, 1.47	0.013	1.76	1.41, 2.19	<0.001
Elixhauser Comorbidity						
<0						
0	0.83	0.73, 0.94	0.003	1.46	1.19, 1.79	<0.001
1 to 4	1.00	0.85, 1.17	0.953	1.48	1.17, 1.87	<0.001
>=5	0.97	0.87, 1.08	0.579	1.72	1.45, 2.04	<0.001
NA	0.94	0.82, 1.09	0.427	1.94	1.52, 2.48	<0.001
Immunocompromised Patient						
No						
Yes	1.14	1.03, 1.27	0.010	1.25	1.11, 1.42	<0.001

Abbreviations: aHR = adjusted hazard ratio, CI = confidence interval.
Model adjusted for age, sex, race, comorbidities, vaccination status, and immunocompromised status.
